# Transcatheter Angiographic Embolization of Percutaneous Nephrolithotomy-Related Bleeding: A Single-Center Experience

**DOI:** 10.1155/2022/4422547

**Published:** 2022-05-17

**Authors:** Fan Xiao, Yang Xun, Weijie Hu, Qidong Xia, Jiaqiao Zhang

**Affiliations:** Department of Urology, Tongji Hospital, Tongji Medical College, Huazhong University of Science and Technology, Wuhan, Hubei 430030, China

## Abstract

**Background:**

To evaluate the clinical characteristics and angiographic features of transcatheter angiographic embolization (TAE) in patients with active bleeding after percutaneous nephrolithotomy (PCNL).

**Methods:**

Between 2009 and 2018, 45 patients who underwent TAE for hemorrhage control after PCNL were reviewed retrospectively. Patient clinical characteristics and angiographic features of TAE were analyzed.

**Results:**

Of the 3148 patients, 45 (1.4%) patients were treated with TAE after PCNL. The interval from the bleeding episode to TAE was 3 days (1,6). Arterial laceration, arteriovenous fistula, and negative angiographic finding were found in 28 (62.2%), 7 (15.6%), and 10 patients (22.2%). Thirty-five patients (92.1%) achieved primary clinical success. The median-corrected hemoglobin decrease from bleeding episode to TAE was 52 g/L (25.25, 71.00). The median-corrected hemoglobin decrease rate from bleeding episode to TAE was 13.11 g/L·d (5.60, 26.12). The hemoglobin decrease from bleeding episode to TAE was lesser in negative angiographic patients (28.50 (10.75,51.25) g/L VS 53.7 (35.0,73.13) g/L) than in positive angiographic patients (*P* < 0.05).

**Conclusions:**

TAE is a safe and effective treatment for post-PCNL bleeding patients. Previous kidney surgery is associated with a higher risk of TAE. Patients with bleeding from multiple negative angiographic findings can be considered for prophylactic embolization.

## 1. Introduction

Percutaneous nephrolithotomy (PCNL) is the first line of treatment for large or complex renal stones [1]. Because of the high stone-free rates, it remained the treatment of choice compared to other minimally invasive modalities. Instead, it is used increasingly for the treatment of renal and ureteral stones [2]. However, some potential complications, such as clinically significant bleeding, can occur. A systematic review reported that the transfusion rates after PCNL ranged between 0% and 20%, with an overall rate of 7%, whereas the embolization rates vary from 0.6% to 1.5% [3]. For severe bleeding after PCNL, transcatheter angiographic embolization (TAE) has been recognized as the preferred treatment [4]. Few studies have reported embolization for delayed hemorrhage after PCNL [5–8]. However, the most suitable timing for performing TAE is still unclear. In addition, few studies have systematically summarized embolization after PCNL. Therefore, this study aimed to evaluate clinical characteristics and angiographic features of TAE in patients with active bleeding after PCNL. Here, we summarize our institutional experience, and provide practitioners a preliminary guidance so that they may best determine which patients could benefit from this treatment approach.

## 2. Material and Methods

### 2.1. Patients

Between October 2009 and April 2018, 3148 patients who underwent PCNL at our institution for kidney stones and 45 patients who underwent TAE for hemorrhage control after PCNL were retrospectively analyzed. Ethical approval was obtained for this retrospective analysis, and the requirement for informed consent was waived. PCNL was performed by experienced urologists using the standardized Chinese percutaneous nephrolithotomy technique developed at our institution [9, 10]. All surgeons were trained at the same institution. History of kidney surgery includes open kidney surgery and PCNL. Sudden onset bleeding was defined as gross hematuria and/or bloody nephrostomy drainage occurring within 24 h after PCNL, whereas that occurring after 24 hours is considered delayed bleeding. The indications for TAE were renal bleeding not controlled by conservative treatments, such as bed rest, tamponade by clamping the nephrostomy tube, hemostatic drugs, and blood transfusion.

Patient demographics of embolization and total PCNL, interval (days) from the bleeding episode to TAE, bleeding characteristics, angiographic findings, number of embolization, embolic material, hemoglobin levels at TAE, hemoglobin and hematocrit decrease during bleeding episode to TAE, TAE to the first post-TAE day, the first post-TAE day to the third post-TAE day, and the number of units transfused were gathered from the medical records. Hemoglobin levels were corrected for any transfusion between the postoperative bleeding episode and TAE. One unit of concentrated red blood cells or whole blood transfused was defined as the equivalent of 10 g/L hemoglobin lost. Two interventional radiologists reviewed the angiographic findings. Vascular lesions were identified based on the type and location of the segmental artery involved.

### 2.2. Transcatheter Angiographic Embolization

After routine disinfection in the groin area, local anesthesia was performed with 2% lidocaine. The femoral artery was punctured by Seldinger technique, and a 5-Fr catheter sheath was placed. Abdominal angiography was performed to evaluate for the presence of renal artery bleeding by using a 5-Fr catheter positioned at the renal artery level. Arteriovenous fistulae (AVF) were characterized by early filling of the venous system during angiography. Arterial laceration was defined as the narrowing of the lumen of the renal artery, with contrast medium spillover. Superselective embolization was performed depending on the angiographic findings. The catheter or microcatheter was placed through the renal artery to approach the lesion as closely as possible, and an embolic material was used for occlusion. The embolic materials consisted of gelatin sponge and metallic coils that were selected during the procedure based on the lesion diameter and type of hemorrhage. Angiography was repeated to verify occlusion of the bleeding point. The catheters were subsequently pulled, and the procedure was terminated. Clinical success was defined as clinical and radiographic absence of bleeding and hemodynamic stability. For patients with negative angiography but clinically highly suspected arterial injury, preventive embolization of the artery near the puncture site was performed. Secondary embolization was performed for persistent bleeding after initial embolization.

### 2.3. Statistical Analysis

All analyses were performed by SPSS software version 24.0. Continuous variables were presented as mean ± standard deviation or median (interquartile range). Statistical analysis was performed with the *t*-test and Mann–Whitney *U* test. Categorical variables were presented as frequencies or percentages, and chi-squared test was performed. The difference was considered statistically significant if *P*-value was <0.05 for all analyses.

## 3. Results

### 3.1. Clinical Characteristics between TAE and Total PCNL

The rate of TAE after PCNL in all patients who underwent PCNL was 1.4% (45/3148), 1.9% (35/1799) among men, 0.7% (10/1349) among women. The rate of TAE was 0.4% (5/1369) between 2008 and 2013, 2.2% (40/1779) between 2014 and 2018) patients.  Table 1 shows clinical characteristics between angiographic embolization and total PCNL. The mean age for angiographic embolization and total PCNL was 53.90 ± 13.06 years and 49.89 ± 11.44 years (*P*=0.056). Furthermore, angiographic embolization was usually associated with longer hospital stay (19.09 ± 10.78 days VS 8.47 ± 3.78 days) and more hospital cost (70158.16 ± 19478.27 yuan VS 35302.62 ± 14366.46 yuan) than normal PCNL (*P* < 0.05).

### 3.2. General Characteristics of TAE

**Figure 1:**
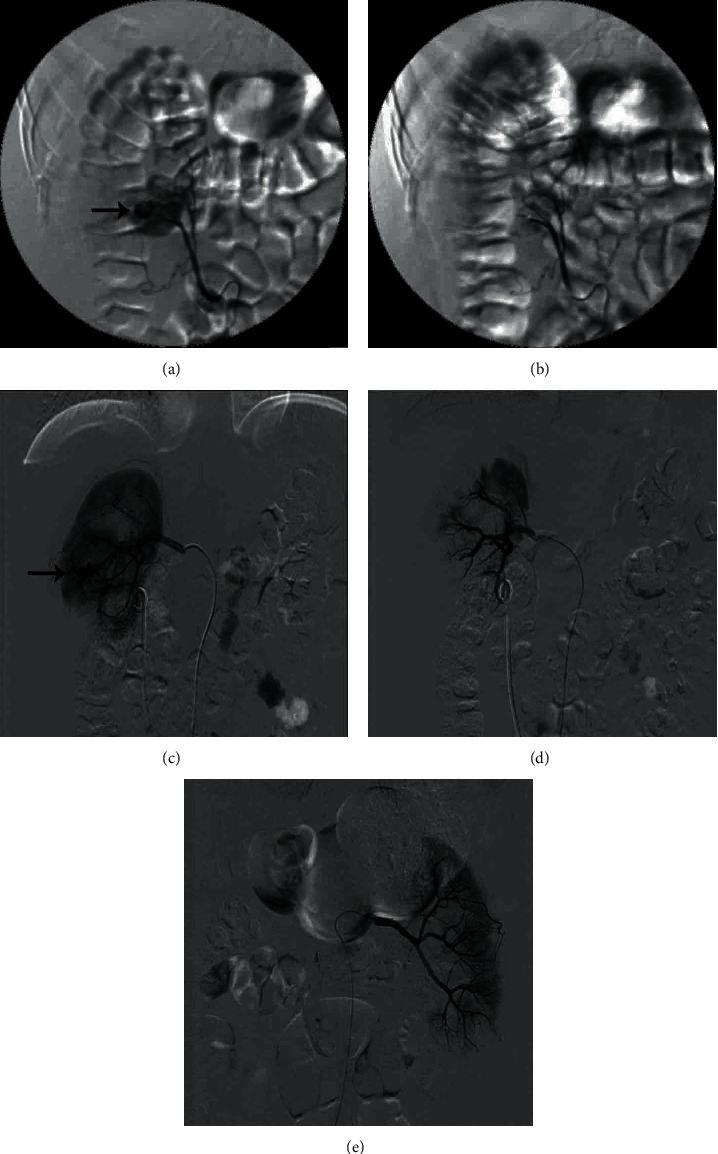
(a) Renal artery branch laceration before superselective TAE. Bleeding location (arrow). (b) Renal artery branch laceration after superselective TAE. (c) Renal arteriovenous fistula before superselective TAE. Bleeding location (arrow). (d) Renal arteriovenous fistula after superselective TAE. (e) No obvious signs of bleeding were found during angiography.

Table 2 summarizes the clinical characteristics, angiographic findings, and results of transarterial embolization. The study included 45 patients with a mean body mass index (BMI) of 24.10 ± 4.14 kg·m^−2^. Twenty-two patients (48.89%) had stones on the left, and 23 (51.11%) patients had stones on the right. Ten patients (22.22%) had a history of kidney surgery. The mean operative time was 103.22 ± 42.21 min. The number of puncture sites in the upper, middle, and lower calyx was 8 (17.78%), 35 (77.78%), and 2 (4.44%), respectively. The interval from the bleeding episode to TAE was 3 days (1–6 days). With regard to bleeding characteristics, 27 patients (60.0%) and 18 patients (40.0%) experienced sudden onset and delayed bleeding. Arterial laceration, AVF, and negative angiographic findings were shown on renal angiography in 28 (62.2%), 7 (15.6%), and 10 (22.2%) patients (Figure 1). Thirty-five patients (92.1%) achieved primary clinical success. A second TAE procedure was required in two patients (4.4%) for uncontrolled persistent bleeding after initial embolization. The first patient underwent a first TAE due to arterial laceration and a second embolization due to bleeding from another renal artery branch. The second patient underwent embolization for arterial laceration and found that there was still bleeding in the same renal artery branch. Prophylactic embolization of the artery near the puncture site was performed in three patients (6.7%) with negative angiography but clinically highly suspected arterial injury; the other seven patients (15.6%) did not undergo embolization due to the negative angiography. The bleeding sites were embolized using gelatin sponge, metallic coils, and combination of metallic coils with gelatin sponge in 7 (15.6%), 3 (6.7%), and 28 patients (62.2%), respectively.

### 3.3. Changes of Hemoglobin and Hematocrit Levels in Patients of TAE

Changes of hemoglobin and hematocrit levels are summarized in Table 3. The median hematocrit decrease from bleeding episode to TAE, TAE to the first post-TAE day, and the first post-TAE day to the third post-TAE day was 10% (6.55, 16.05), 2.8% (1.45, 4.85), and 1.9% (0.83, 3.45), respectively. The median minimum hemoglobin level before embolization was 89 g/L (72.48, 106). The mean-corrected hemoglobin decrease from bleeding episode to TAE, TAE to the first post-TAE day and the first post-TAE day to the third post-TAE day was 52 g/L (25.25, 71.00), 10 g/L (−1, 21) and 3.5 g/L (−2.75, 11.00), respectively. The median-corrected hemoglobin decrease rate from bleeding episode to TAE and TAE to the first post-TAE day was 13.11 g/L d (5.60, 26.12) and 6.0 g/L d (−1.5, 10.75), respectively.

### 3.4. Comparison between Negative Angiography and Positive Angiography

Subgroup analysis was performed to compare patients with negative and positive angiography findings (Table 4). Sex, age, BMI, SSD, history of kidney surgery, and bleeding characteristics were not significantly different between the two groups (*P* > 0.05). The patients with negative angiography findings had higher minimum hemoglobin level before embolization [107 (87.5,124.5) g/L VS 78.5 (69.75, 99.5) g/L], lower hematocrit decrease [6.9% (1.93, 8.98) VS 11.95 (7.68,17.60)], and hemoglobin decrease [28.50 g/L (10.75, 51.25) VS 53.7 g/L (35.0, 73.13)] from bleeding episode to TAE than patients with positive angiographic findings (*P* < 0.05). The hemoglobin decrease rate in patients with negative angiography [9.65 g/L·d (6.25, 20.25)] was slower than patients with positive angiographic findings [13.52 g/L·d (5.16, 28.12)], although it is not statistically significant.

## 4. Discussion

Compared with the other minimal invasive lithotripsy techniques, PCNL is generally considered as a safe technique offering the highest stone-free rates [11]. However, serious complications still occur following this percutaneous procedure. Severe bleeding requiring transfusion is a relatively rare complication associated with PCNL. Most bleeding complications can be well controlled with conservative treatments [12]. The post-PCNL transfusion rates ranging between 1% and 55% have been documented in recent studies. The use of selective renal artery angiography and embolization has ranged from 0.6% to 1.5% [3, 13]. In our study, 45 (1.4%) of 3148 patients underwent TAE following PNL. This rate is in accordance with the literature.

In this study, we found that patients requiring TAE after PCNL had twice hospital stays and hospitalization costs than patients without complications, and it is statistically significant. This was not reported in the previously related studies. Patients requiring TAE after PCNL have a high proportion of previous kidney surgery (22.22%) in our results. A recent meta-analysis also shows that PCNL in patients with previous open renal surgery is associated with a significantly higher risk of requiring TAE [14]. Possible reason may be that previous kidney surgery is usually associated with anatomical alterations, inflammation, and adhesions. Performing PCNL at this site could lead to vascular problems. Furthermore, retroperitoneal and caliceal scarring surrounding the kidney may reduce its mobility [15]. Therefore, intraoperative manipulation of the nephroscope may produce enough torque to cause lacerations to the kidney with bleeding. Performing PCNL should be more cautious for patients with previous kidney surgery.

There is currently no standard classification for postoperative bleeding from PCNL. Based on the International Study Group of Pancreatic Surgery and Liver Surgery [16, 17], early bleeding was defined as post-hemorrhage occurring within the first 24-h postoperatively, whereas >24 h is considered delayed bleeding. Vascular injury during surgical procedures or underlying perioperative coagulopathy may be the main cause of early post-PCNL bleeding. Delay post-PCNL bleeding is typically a surgical complication, with the usual delay from days to weeks [6]. Post-PCNL bleeding is usually caused by pseudoaneurysm, AVF, and arterial laceration in the previously related studies. When renal arterial bleeding does occur, blood from the injured artery can leak freely due to the high pressure, drain into the injured adjacent vein resulting in AVF, or into renal parenchyma or hilar areolar tissue resulting in pseudoaneurysm [5, 6, 18]. However, in our cases, these life-threatening hemorrhages are usually caused by arterial laceration (28, 62.20%) and AVF (7, 15.60%). The main reason is that our radiologist did not report pseudoaneurysm directly, but attributed it to arterial laceration. This study evaluated the therapeutic effect of TAE on post-PCNL bleeding. Successful embolization was performed in all 38 patients, including 35 with vascular lesions and three prophylactic embolizations. Primary clinical success was 94.3% (33/35), and only two patients developed second TAE owing to uncontrolled persistent bleeding after initial embolization. With regard to secondary embolization, one patient showed different bleeding sites in the two angiographic results, and another patient showed bleeding at the first embolization site. One patient did not find bleeding site in the two angiographic results, another two patients with clinically highly suspected arterial injury; hence, prophylactic embolization was performed. In most cases, the success rate of embolization is high, but a small number of patients may need secondary embolization because of the poor first embolization effect or undetected bleeding site. For patients with negative first-time angiography, most of the bleeding was gradually stable, no further embolization was needed, and only one case continued to bleed. No bleeding was found after re-angiography; hence, prophylactic embolization was performed on the artery near the puncture site. We recommend that patients with bleeding from multiple negative angiographic findings can be considered for prophylactic embolization.

For urologists, uncontrolled bleeding after PCNL is a stressful complication. The selection of continued conservative treatment or TAE can be difficult. Therefore, a clear indication for TAE is very important. Oguz et al. recommended that emergent surgical intervention should be performed if metabolic acidosis and anuria/oliguria accompanied the decrease of hemoglobin [19]. Jinga et al. [20] reported that the variations of hemoglobin, together with the quantity of transfused units, are an indication for TAE. However, these indications lack specific indices and have not been validated. Currently, there are still a lack of strict indications for TAE in post-PCNL bleeding. Hemoglobin decrease is the most common indicator, which is used to assess the severity of bleeding in clinical surgery [21, 22]. Li et al. [6] proposed that TAE should be the recommended treatment for delayed post-PCNL hemorrhage in patients with hemodynamic instability and/or corrected hemoglobin decrease of >30 g/L following conservative management. However, relying solely on the hemoglobin decrease to determine the timing of TAE is not adequate. For some patients, hemoglobin decrease may be not obvious, but it occurs in a short time. This situation also requires timely TAE. Hence, we proposed the concept of hemoglobin decrease rate. In our study, we calculated the changes in hemoglobin and hematocrit during bleeding episode to TAE, TAE to the first post-TAE day, and the first post-TAE day to the third post-TAE day, respectively. The decrease of hemoglobin/hematocrit and hemoglobin decrease rate was significantly reduced after TAE therapy, suggesting a good treatment effect. Moreover, we found that some patients still had minimal bleeding after TAE, but most tend to be stable within 1–3 days. Based on these findings, we recommend that hemoglobin decrease of >25 g/L or hemoglobin decrease rate of >5.5 g/L·d following conservative management of bleeding as one of the indications for TAE.

In our study, 10 patients had negative angiographic findings. Choi et al. [8] reported that angiographic findings were not significantly associated with age, sex, and PCNL, and only percutaneous nephrostomy was associated with a higher rate of negative angiographic findings. Our subgroup analysis also shows similar results. However, we found that the decrease of hemoglobin/hematocrit levels in patients with negative angiographic finding was significantly lower than that in patients with positive angiographic finding. Patients with negative angiographic findings also had a lower hemoglobin decrease rate. The minimal bleeding rate required for angiographic detection is 0.5 mL/min. Angiography becomes optimally sensitive when the bleeding rate reaches 1 mL/min, which is equivalent to three units per day [23]. Slow bleeding rate may be one of the reasons of negative angiographic findings. Vasospasm or minor vascular laceration is also an important factor [6]. Vasospasm may occasionally account for a negative result shortly after bleeding [24]. In addition, selective angiography is limited in showing venous bleeding. For the patients with negative angiographic findings, post-PCNL bleeding may be controlled by conservative treatment; hence, embolization may be worth considering. More research is needed to further explore the timing of embolization for these patients.

The present study had some limitations. First, it was a single-center retrospective study, which makes obtaining relevant information difficult. Second, our radiologist did not directly report pseudoaneurysm, but attributed it to arterial laceration. Another limitation was this study lacks control and randomization. Multi-center studies with larger samples and longer follow-up periods are needed for further validation. In conclusion, TAE is a safe and effective treatment for patients with post-PCNL bleeding. Previous kidney surgery is associated with a higher risk of TAE. Patients with bleeding from multiple negative angiographic findings can be considered for prophylactic embolization.

## Figures and Tables

**Table 1:** Clinical characteristics between angiographic embolization and total PCNL.

	Angiographic embolization	Total PCNL	*p*
Patients (n)	45	3148	
Generation (y) , n (%)			
2009–2013	5 (0.4%)	1369	
2014–2018	40 (2.2%)	1779	
Sex, n (%)			<0.005
Male	35 (77.78%)	1799 (57.15%)	
Female	10 (22.22%)	1349 (42.85%)	
Age (y), mean (SD)	53.90 ± 13.06	49.89 ± 11.44	0.056
Hospital stay (d), mean (SD)	19.09 ± 10.78	8.47 ± 3.78	<0.05
Hospital cost (y), mean (SD)	70158.16 ± 19478.27	35302.62 ± 14366.46	<0.05

**Table 2:** Clinical characteristics, angiographic findings, and results of transarterial embolization.

Patients (n)	45

BMI (kg m^−2^), mean (SD)	24.10 ± 4.14
Stone laterality, n (%)	
Left	22 (48.89%)
Right	23 (51.11%)
History of kidney surgery, n (%)	
Yes	10 (22.22%)
No	35 (77.78%)
Operation time (min), mean (SD)	103.22 ± 42.21
Puncture calyx, n (%)	
Upper	8 (17.78%)
Mid	35 (77.78%)
Lower	2 (4.44%)
Intervals (days) from the bleeding episode to TAE Median (IQR)	3 (1–6)
Bleeding site, n (%)	
Upper pole	2 (4.40%)
Mid-pole	14 (31.10%)
Lower pole	12 (26.70%)
Mid-pole + lower pole	1 (2.20%)
Undefined	16 (35.60%)
Bleeding characteristics, n (%)	
Sudden onset bleeding	27 (60.0%)
Delayed bleeding	18 (40%)
Angiographic findings, n (%)	
Arterial laceration	28 (62.20%)
Arteriovenous fistula	7 (15.60%)
Negative angiographic finding	10 (22.20%)
Number of embolization, n (%)	
No embolization	7 (15.60%)
Prophylactic embolization	3 (6.7%)
One-time embolization	33 (73.30%)
Two-times embolization	2 (4.4%)
Results of transarterial embolization, n (%)	
Primary clinical success rate	35 (92.10%)
Secondary clinical success rate	38 (100%)
Embolic material, n (%)	
Gelatin sponge	7 (15.60%)
Metallic coils	3 (6.70%)
Metallic coils + gelatin sponge	28 (62.20%)
No embolization	7 (15.60%)

**Table 3:** Changes in hemoglobin and hematocrit.

Hematocrit decrease (g/L), median (IQR)	
Bleeding episode to TAE	10 (6.55, 16.05)
TAE to D1	2.8 (1.45, 4.85)
D1 to D3	1.9 (0.83, 3.45)

Minimum hemoglobin before embolization (g/L), median (IQR)	89 (72.48, 106)

Hemoglobin decrease (g/L), median (IQR)	
Bleeding episode to TAE	52 (25.25, 71.00)
TAE to D1	10 (−1, 21)
D1 to D3	3.5 (−2.75, 11.00)

Hemoglobin decrease rate (g/L·d), median (IQR)	
Bleeding episode to TAE	13.11 (5.60, 26.12)
TAE to D1	6.0 (−1.5, 10.75)

TAE, transcatheter angiographic embolization; D1, the first post-TAE day; D3, the third post-TAE day.

**Table 4:** Comparison between negative angiography and positive angiography.

	Negative angiography	Positive angiography	*P*
Patients (n)	10	35	
Sex, n (%)			0.533
Male	9 (90%)	26 (74.29%)	
Female	1 (10%)	9 (25.71%)	
Age (y), mean (SD)	49.80 ± 14.85	52.51 ± 12.30	0.560
BMI (kg m^−2^), mean (SD)	23.96 ± 4.05	24.14 ± 4.23	0.907
History of kidney surgery, n (%)			0.533
Yes	1 (10%)	9 (25.71%)	
No	9 (90%)	26 (74.29%)	
Bleeding characteristics, n (%)			0.714
Sudden onset bleeding	5 (50%)	22 (62.86%)	
Delayed bleeding	5 (50%)	13 (37.14%)	
Patients with transfusion before TAE, n (%)			0.811
Yes	5 (50%)	16 (45.7%)	
No	5 (50%)	19 (54.3%)	
Minimum hemoglobin before embolization (g/L), median (IQR)	107 (87.5,124.5)	78.5 (69.75,99.5)	0.013
Bleeding episode to TAE, Median (IQR)			
Hematocrit decrease (g/L)	6.9 (1.93,8.98)	11.95 (7.68,17.60)	0.007
Hemoglobin decrease (g/L)	28.50 (10.75,51.25)	53.7 (35.0,73.13)	0.019
Hemoglobin decrease rate (g/L·d)	9.65 (6.25,20.25)	13.52 (5.16,28.12)	0.779

## Data Availability

The data that support the findings of this study are available on request from the corresponding author. The data are not publicly available due to privacy or ethical restrictions.

## Abbreviations

TAE: Transcatheter angiographic embolization
PCNL: Percutaneous nephrolithotomy
AVF: Arteriovenous fistulae
BMI: Body mass index.
